# Ablation of RIP3 protects from dopaminergic neurodegeneration in experimental Parkinson’s disease

**DOI:** 10.1038/s41419-019-2078-z

**Published:** 2019-11-05

**Authors:** Pedro A. Dionísio, Sara R. Oliveira, Maria M. Gaspar, Maria J. Gama, Margarida Castro-Caldas, Joana D. Amaral, Cecilia M. P. Rodrigues

**Affiliations:** Research Institute for Medicines (iMed.ULisboa), Faculty of Pharmacy, Universidade de Lisboa, Lisbon, Portugal

**Keywords:** Parkinson's disease, Chronic inflammation

## Abstract

Parkinson’s disease (PD) is driven by dopaminergic neurodegeneration in the *substantia nigra pars compacta* (SN) and striatum. Although apoptosis is considered the main neurodegenerative mechanism, other cell death pathways may be involved. In this regard, necroptosis is a regulated form of cell death dependent on receptor interacting protein 3 (RIP3), a protein also implicated in apoptosis and inflammation independently of its pro-necroptotic activity. Here, we explored the role of RIP3 genetic deletion in in vivo and in vitro PD models. Firstly, wild-type (Wt) and RIP3 knockout (RIP3ko) mice were injected intraperitoneally with MPTP (40 mg/kg, i.p.), and sacrificed after either 6 or 30 days. RIP3ko protected from dopaminergic neurodegeneration in the SN of MPTP-injected mice, but this effect was independent of necroptosis. In keeping with this, necrostatin-1s (10 mg/kg/day, i.p.) did not afford full neuroprotection. Moreover, MPTP led to DNA fragmentation, caspase-3 activation, lipid peroxidation and BAX expression in Wt mice, in the absence of caspase-8 cleavage, suggesting intrinsic apoptosis. This was mimicked in primary cortical neuronal cultures exposed to the active MPTP metabolite. RIP3 deficiency in cultured cells and in mouse brain abrogated all phenotypes. Curiously, astrogliosis was increased in the striatum of MPTP-injected Wt mice and further exacerbated in RIP3ko mice. This was accompanied by absence of microgliosis and reposition of glial cell line-derived neurotrophic factor (GDNF) levels in the striata of MPTP-injected RIP3ko mice when compared to MPTP-injected Wt mice, which in turn showed a massive GDNF decrease. RIP3ko primary mixed glial cultures also presented decreased expression of inflammation-related genes upon inflammatory stimulation. These findings hint at possible undescribed non-necroptotic roles for RIP3 in inflammation and MPTP-driven cell death, which can contribute to PD progression.

## Introduction

Parkinson’s disease (PD) is the second most common neurodegenerative disorder and its prevalence is expected to increase^1^. Pathologically, PD is characterized by progressive dysfunction of the nigrostriatal pathway, ultimately leading to loss of dopaminergic enervation in the striatum and concomitant neurodegeneration in the *substantia nigra* *pars compacta* (SN)^1,2^. As most chronic neurodegenerative disorders, PD aetiology is complex, involving oxidative damage, mitochondrial dysfunction, inflammation and neuronal death. Unfortunately, generalized loss of dopaminergic neurons in the SN precedes the appearance of the first PD motor symptoms^2^. This feature of PD, allied to its multifactorial aetiology, has hindered this far the development of suitable therapies against disease progression^1^.

The causes of neuronal death in PD are still a matter of controversy. Apoptosis has been considered the main mechanism of neuronal demise in most neurodegenerative conditions, including PD^3–5^, but other regulated cell death (RCD) pathways are now emerging that are likely to contribute to neurodegeneration in the disease^3,6,7^. Of note, necroptosis, a form of regulated cell death that macroscopically mimics necrosis, has been recently implicated in neuronal death caused by the PD-mimicking neurotoxin 1-methyl-4-phenyl-1,2,3,6-tetrahydropyridine (MPTP) in an in vivo mouse model, while some landmarks of this RCD have been detected in human PD samples^7^. In fact, necroptosis has been described in several neurodegenerative disorders, all of them linked by strong neuroinflammatory features, further supporting a role for this RCD in PD^8–10^.

Necroptosis is a caspase-independent form of RCD usually executed following the activation of the kinase activities of receptor interacting protein (RIP) 1 and RIP3. Necroptosis is typically activated after the stimulation of a transmembrane receptor mainly linked to cell death and/or inflammation, such as the tumour necrosis factor α (TNF-α) receptor 1 (TNFR1)^11,12^. Receptor stimulation then leads to the recruitment of a multiprotein platform which includes RIP1, culminating in the formation of pro-survival complex I^11^. Depending on post-translational modifications controlled by cellular context, RIP1 can signal for cell survival/inflammation, apoptosis or necroptosis. Dissociation of RIP1 from complex I may lead to the formation of the cytosolic complex II (or Ripoptosome), along with Fas-associated protein with death domain (FADD) and caspase-8, which activates caspase-8 for the induction of extrinsic apoptosis^11^. Active caspase-8 then cleaves RIP1 and RIP3, which abrogates subsequent interactions between these two proteins in complex II and, therefore, necroptosis. However, if caspase-8 is inhibited or absent, RIP1 and RIP3 may further oligomerize through their RIP homotypic interaction motif (RHIM)-domains, leading to auto-phosphorylations that allow the formation of an insoluble amyloid-like structure named necrosome^11,13^. Here, RIP3 phosphorylates mixed lineage kinase domain-like protein (MLKL) at S345 in mouse, a key event for necroptosis by allowing MLKL oligomerization and translocation to cellular membranes, with subsequent cell permeabilization^11,14^. However, several lines of evidence indicate that RIP3 may have multiple roles in cell death and inflammation, which are independent of its pro-necroptotic activity. For example, RIP3 may accelerate Ripoptosome assembly and caspase-8 activation, thus facilitating apoptosis^15^. RIP3 can also contribute to inflammasome activation in a MLKL-independent fashion and modulates TLR-downstream signalling at least in some settings^16,17^.

Here, RIP3 deletion protected from dopaminergic neurodegeneration in the SN of a sub-acute MPTP mouse model of PD, while replenishing glial cell-line derived neurotrophic factor (GDNF) protein levels in the striatum. Surprisingly, necroptosis remained undetected in in vivo and in vitro experiments following exposure to MPTP or its toxic metabolite 1-methyl-4-phenylpyridinium (MPP^+^). Conversely, our results suggest activation of mitochondrial-dependent intrinsic apoptosis, which was abolished by RIP3 deficiency. Moreover, RIP3 ablation dampened the inflammatory response in primary mixed glial cultures, supporting non-necroptotic roles for RIP3.

## Materials and methods

### MPTP animal models

All animal experiments were conducted according to the animal welfare organ of the Faculty of Pharmacy, University Lisbon, approved by the competent national authority Direção-Geral de Alimentação e Veterinária (DGAV) and in accordance with the EU Directive (2010/63/UE), Portuguese laws (DR 113/2013, 2880/2015 and 260/2016) and all relevant legislation. To evaluate the role of RIP3 in the sub-acute MPTP mouse model, we used male 13-week-old C57BL/6N wild-type (Wt) (Charles River Laboratories, Wilmington, MA, USA) and RIP3 knockout (RIP3ko) mice generated on the same C57BL/6N background (Genentech, South San Francisco, CA, USA)^18^. Mice were injected intraperitoneally (i.p.) with a single dose of MPTP-HCl (40 mg/kg; Sigma Aldrich, St Louis, MO, USA), dissolved in sterile 0.9% saline, or vehicle alone—control group (seven animals/group)^19,20^. Following 30 days after neurotoxin or vehicle injection, mice were sacrificed in a CO_2_ chamber followed by transcardiac perfusion with ice-cold phosphate buffered saline (PBS). Then, brains were excised, and one hemisphere was fixed in in 4% paraformaldehyde for 48 h and stored in 20% sucrose/PBS + 0.025% sodium azide at 4 °C for posterior immunohistochemistry analyses. The other hemisphere was used to isolate the midbrain region, containing the SN, and the striatum, as previously described, which were then flash frozen and stored at −80 °C until further processing for protein extraction^20^. Wt animals were also sacrificed at 4 and 6 days after MPTP or vehicle injection (four animals/group) to search for necroptotic markers at these time-points. RIP3 deletion was confirmed in RIP3ko mice by genotyping following tail biopsies and DNA extraction by conventional PCR with a mixture of the following primers: 1- 5′-CGCTTTAGAAGCCTTCAGGTTGAC; 2- 5′-GCAGGCTCTGGTGACAAGATTCATGG; 3- 5′-CCAGAGGCCACTTGTGT AGCG, which yields a 700 bp electrophoretic band (Wt Rip3 allele) or a 450 bp band (Rip3 deletion allele).

Furthermore, to determine whether pharmacological inhibition of RIP1-dependent necroptosis could be protective in our MPTP mouse model, Wt mice were injected i.p. with 10 mg/kg necrostatin-1s (Nec-1s) (Focus Biomolecules, Plymouth Meeting, PA, USA) solubilized in DMSO 1%, 2-hydroxypropyl-beta-cyclodextrin 4% (both from Sigma Aldrich) in PBS, 1 h after MPTP administration, and once every day for 30 days as aforementioned. Nec-1s dosage and regimen of administration were selected according to published protocols^7,8^.

### Pole test

Mice were placed at the top of a pole (diameter, 1 cm; height 55 cm) facing upwards, and the time spent turning around and climbing down the pole was recorded, according to previous protocols^21,22^. Each mouse performed for 3 runs and the average time was calculated.

### Primary cultures

Primary neuronal cultures were prepared as previously described^23^. Briefly, C57BL/6 pregnant mice at gestational days 17–18 were sacrificed in a CO_2_ chamber and the fetuses were quickly collected in Hank’s balanced salt solution (HBSS) (Gibco^TM^, Thermo Fisher Scientific Inc., Waltham, MA, USA) and decapitated. Brains were collected and, after removal of meninges, cortices were isolated and mechanically fragmented. Then, the fragments were incubated in a 0,05% trypsin solution (Sigma Aldrich) at 37 °C for 15 min with agitation. After, cells were resuspended in HBSS supplemented with 10% heat-inactivated fetal bovine serum (FBS) (Gibco^TM^) and further dissociated by gentle pipetting, followed by two washing cycles at 2000 rpm and resuspension in the same solution as before. After the last centrifugation, cells were resuspended in Neurobasal medium, supplemented with 0.5 mM l-glutamine, 2% B-27 supplement, 12 mg/ml gentamicin (Gibco™) and 25 μM l-glutamic acid (Sigma Aldrich). Then, cells were plated at 6.4 × 10^5^ cells/cm^2^ on culture plates pre-coated with poly-D-lysine (Sigma Aldrich), and maintained in a humidified atmosphere of 5% CO_2_ at 37 °C. Half the medium of the primary cortical neuronal cultures were changed every 3–4 days until the 7^th^ day in vitro (7DIV).

Primary mixed cultures were prepared as previously described^24^. Briefly, meninge-free cortices from 2–3-days-old C57BL/6 mouse pups were isolated in HBSS and the tissue was mechanically dissociated by pipetting followed by sequential passages through steel screens of 230-, 104- and 74-μm pore sizes (Sigma Aldrich). Isolated cells were plated at 4 × 10^5^ cells/cm^2^ in cultures plates in DMEM-F12 + GlutaMAX^TM^ medium supplemented with 10% FBS and 1% antibiotic/antimycotic (all from Gibco™) and maintained in a humidified atmosphere of 5% CO_2_ at 37 °C. Medium was changed at 7DIV and primary mixed glial cultures were used at 10DIV, when they were nearly confluent. Microglia accounted for approximately 10% of the cells in the culture, as determined by ionized calcium binding adaptor molecule 1 (Iba-1) immunostaining.

The reagents used for cell treatments were as follows: MPP^+^ iodide (#D048, Sigma Aldrich), staurosporine (#S4400, Sigma Aldrich), lipopolysaccharide (LPS) from *Escherichia coli* 055:B5 (#437625, Calbiochem - Merck, Darmstadt, Germany), recombinant murine TNF-α (#315-01A, PeproTech EC Ltd., London, UK), recombinant murine interferon-γ (IFNγ) (#315-05, Peprotech).

### Immunohistochemistry

Paraformaldehyde-fixed hemispheres were further cryoprotected in 30% sucrose/PBS and embedded in gelatin. Sequential coronal brain sections (8-μm thick) near the midstriatum (Bregma 1.00) and SN (Bregma −3.20) were obtained by cryostat sectioning and mounted on SuperFrost-Plus glass slides (Thermo Fisher Scientific). To remove gelatin, the sections were incubated in warm PBS at 37 °C for 15 min, followed by two washes in PBS. The sections were then blocked for 1 h in Tris buffered saline (TBS) containing 10% (v/v) normal donkey serum (Jackson ImmunoResearch Laboratories Inc., West Grove, PA, USA) and 0.1% (v/v) Triton X-100 (Sigma-Aldrich). Subsequently, the sections were incubated in appropriately diluted primary antibodies overnight at 4 °C. After several PBS washes, the primary antibodies were detected with diluted (1:200) Alexa Fluor 568 (anti-mouse) or Alexa Fluor 488 (anti-rabbit) conjugated secondary antibodies (Invitrogen - Thermo Fisher Scientific) for 2 h at room temperature. After extensive rinsing, the sections were counterstained with Hoechst 33258 (Sigma-Aldrich) and mounted on Mowiol 4-88 (Sigma-Aldrich). The following primary antibodies were used: rabbit polyclonal anti-tyrosine hydroxylase (TH) was used to stain dopaminergic neurons (#ab112; Abcam, Cambridge, United Kingdom; 1:700); mouse monoclonal anti-glial fibrillary acidic protein (GFAP) was used to stain astrocytes (GA5; #MAB360, Millipore Corporation, Temecula, CA, USA; 1:200); rabbit polyclonal anti-Iba-1 antibody was used to stain microglia (Wako Pure Chemicals, Richmond, VA, USA; 1:100).

### Image analysis

All images were captured using an Axioskop fluorescence microscope (Carl Zeiss GmbH, Hamburg, Germany). Images from six region-matched sections were acquired for nigral and striatal regions for each animal and converted to gray scale with an 8-bit format using the ImageJ software (National Institute of Health, Bethesda, USA). For each staining, a threshold optical density was determined and held constant. Areas occupied by positive staining were then quantified in thresholded images, which was normalized to the total area of the region of interest, being presented as percentage of the total area.

### Terminal deoxynucleotidyl transferase-dUTP nick end labeling (TUNEL) assay

After gelatin removal, coronal brain sections containing SN were processed for detections of DNA fragmentation, a typical apoptotic feature, with the ApopTag® Fluorescein In Situ Apoptosis Detection Kit (#S7710, Millipore), according to the manufacturer’s instructions. After, the sections were counterstained with Hoechst 33258 (Sigma-Aldrich), for nuclei visualization, and mounted on Mowiol 4-88 (Sigma-Aldrich). Images from three region-matched sections at the SN for each animal were acquired in an Axioskop fluorescence microscope (Carl Zeiss) and numbers of apoptotic nuclei were counted.

### Protein isolation

For soluble and insoluble protein extraction, dissected midbrains and striata were processed as described elsewhere^8^. Basically, brain tissues were homogenized in radio-immunoprecipitation assay (RIPA) buffer (50 mM Tris/HCl, pH 8; 150 mM NaCl; 1% NP-40; 0.5% sodium deoxycholate; 0.1% SDS) and 1 × Halt Protease and Phosphatase Inhibitor Cocktail (Pierce – Thermo Fisher Scientific) with a motor-driven Bio-vortexer (No1083; Biospec Products, Bartlesfield, UK). The homogenates were centrifuged at 15,000 g for 20 min at 4 °C, and the resulting supernatants were used as the soluble fraction. The pellets, containing the insoluble protein fractions, were further resuspended in RIPA buffer containing 8 M urea and then sonicated in an ultrasonic processor UP100H (Hielscher Ultrasonics GmbH, Teltow, Germany). For total protein isolation, tissues were homogenized in RIPA buffer as aforementioned, while cell cultures were directly scraped in the same buffer after being washed once in PBS. Then the lysates remained on ice for 30 min, followed by sonication and centrifugation at 10,000 g for 10 min. The supernatant was used as the total protein extract. Protein concentrations were determined using the Bio-Rad protein assay kit, according to the manufacturer’s recommendations.

### Western blot (WB)

Equal amounts of protein (20–50 μg) were resolved on 8% or 12% SDS-PAGE. The resolved proteins were transferred onto nitrocellulose membranes and blocking was performed with a 5% milk solution in TBS. Membranes were then incubated overnight at 4 °C with the following primary antibodies. These included mouse monoclonal: GFAP (GA5), #MAB360, Millipore; B-Cell Leukemia/Lymphoma-2 (Bcl-2) (C-2), #sc-7382; Bcl-2-associated X protein (BAX) (B-9), #sc-7480; GDNF (B-8), #sc-13147; TNF-α (52B83), #sc-52746; caspase-8 (D-8), #sc-5263, Santa Cruz Biotechnology Inc., Dallas, TX, USA; NLRP3 (Cryo-2), #AG-20B-0014-C100, Adipogen Corporation, San Diego, USA; rabbit monoclonal: RIP1 (D94C12), #3493, Cell Signaling, Danvers, MA, USA; rabbit polyclonal: TH, #ab112; 4-Hydroxynonenal (4-HNE), #ab46545; p-MLKL (Ser358), #ab196436, Abcam; caspase-3, active (cleaved) form, #AB3623, Millipore; MLKL, #SAB1302339, Sigma-Aldrich; Voltage-dependent anion channel (VDAC), #4866, Cell Signaling.

After washing with TBS/0.2% Tween 20 (TBS-T), the membranes were incubated with goat secondary antibodies anti-mouse or anti-rabbit conjugated with horseradish peroxidase (BioRad Laboratories, Hercules, CA, USA) for 2 h at room temperature. After rinsing with TBS-T, the immunoreactive proteins were visualized with Immobilon^TM^ Western (Millipore) or SuperSignal West Femto substrate (Thermo Fisher Scientific). β-actin (AC-15), #A5441, Sigma-Aldrich, was used as loading control. Densitometric analyses were performed with the Image Lab software Version 5.1 Beta (Bio-Rad).

### Neuronal cell death and viability

Primary neurons at 7DIV were incubated with increasing concentrations of MPP^+^ or 100 nM staurosporine for 24 h, and total ATP levels were measured with the CellTiter-Glo® Luminescent Cell Viability Assay Kit, while cellular viability and toxicity were respectively determined with the GF-AFC and bis-AAF-R110 Substrates, according to the manufacturer’s specifications (all from Promega Corporation, Madison, WI, EUA).

### Real-Time PCR

After treatment with 100 ng/ml LPS, 50 ng/ml TNF-α, 100 U/ml IFNγ or TNF-α + IFNγ for 24 h, primary mixed glial cultures were scraped in RiboZol™ (VWR Life Science AMRESCO ®, Radnor, PA, USA), and total RNA was isolated according to the manufacturer’s protocol. Total RNA was quantified in a Qubit^TM^ 2.0 fluorometer (Invitrogen – Thermo Fisher Scientific) and converted into cDNA using NZY First-Strand cDNA Synthesis Kit (NZYTech, Lisbon, Portugal), according to the manufacturer’s instructions. Quantitative Real-time PCR (qRT-PCR) analyses were performed in a QuantStudio 7 Flex Real-Time PCR System (Thermo Fisher Scientific), in a final volume of 5 μL using SensiFAST™ SYBR® Hi-ROX Kit (Bioline, Meridian Bioscience, Inc., Cincinnati, OH, USA) and 0.3 μM of each primer pair. The expression levels of the genes of interest relative to the housekeeping gene Hypoxanthine-guanine phosphoribosyltransferase (HPRT) were calculated using the relative standard curve method. Primer sequences are presented in Supplementary Table 1.

### Statistical analysis

Data comparisons were conducted with one-way analysis of Variance (ANOVA) followed by post hoc Bonferroni’s test. Values of *P* < 0.05 were considered statistically significant. Analyses and graphical presentation were performed with the GraphPad Prism software Version 5 (GraphPad Software Inc., San Diego, CA, USA). Results are presented as mean ± standard error of the mean (SEM).

## Results

### Genetic ablation of RIP3 attenuates MPTP-driven dopaminergic neurodegeneration in the SN and striatum

**Fig. 1:**
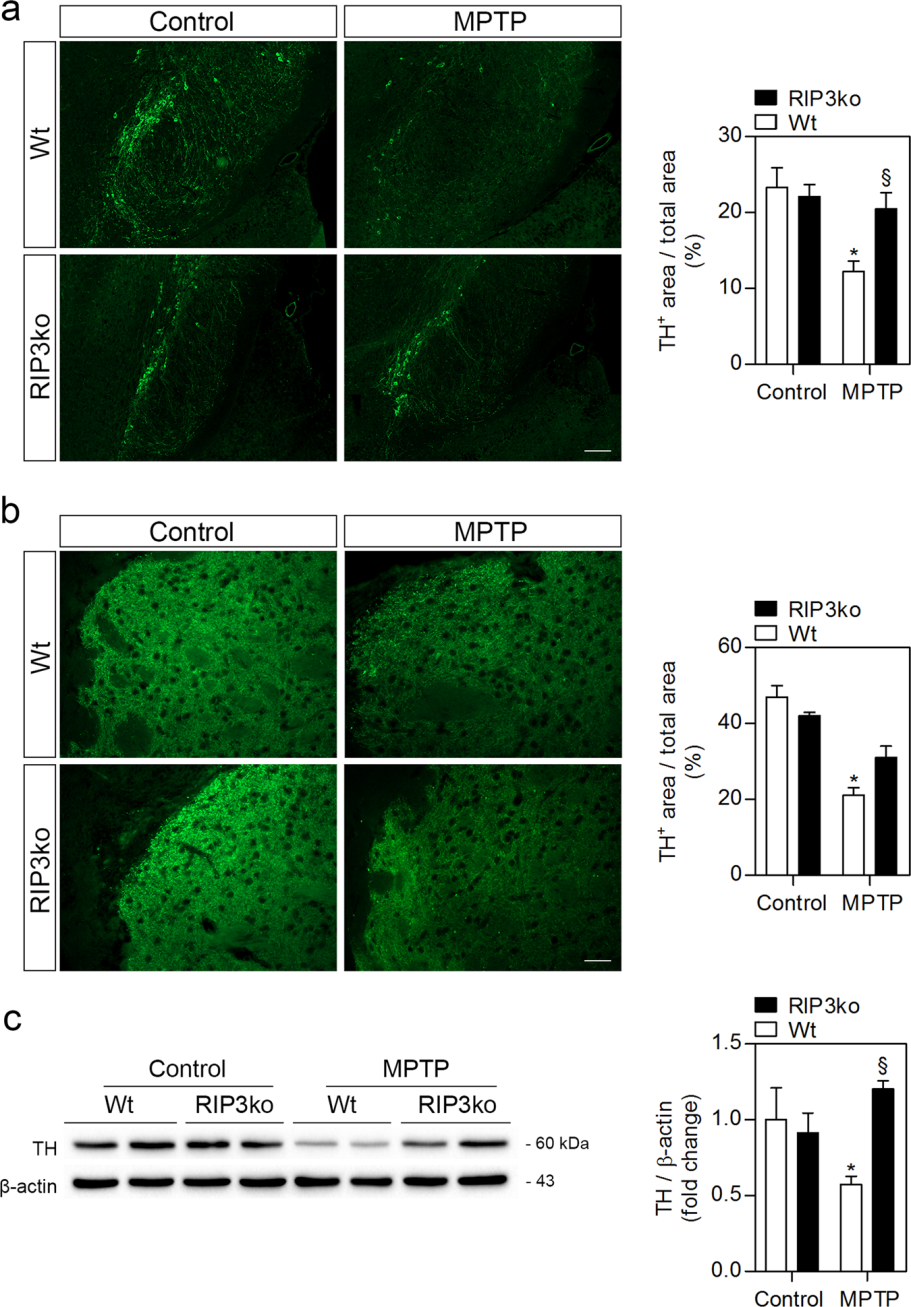
RIP3ko protects from MPTP-driven dopaminergic neurodegeneration. **a** Representative images of TH-positive immunostaining in the SN from control- and MPTP-injected mice from both genotypes and respective quantification. Scale bar, 100 μm. **b** Representative images of TH-positive fibers in the striatum from control- and MPTP-treated mice from both genotypes and respective quantification. Scale bar, 100 μm. **c** Representative WB of TH protein levels in the SN of control- and MPTP-injected Wt and RIP3ko mice and respective densitometric analysis. Values represent mean ± SEM of 6–7 mice per group. *p < 0.05 from control Wt mice and ^§^*p* < 0.05 from MPTP-injected Wt mice

No signs of MPTP-driven bradykinesia were detected in any MPTP-treated groups, as measured by the pole test (data not shown). This test was mostly chosen due to its sensitivity for impaired exercise of fine motor skills, which can highlight subtle motor deficits^25^. However, the impact in motor performance following MPTP administration in mice is controversial, ranging from no observable phenotypes to stable or transient alterations, which depend on MPTP dosage, time after exposure and animal strain and age^25,26^. Here, despite absence of visible motor deficits, MPTP treatment led to a ~50% loss of TH-positive staining in the SN (Fig. 1a, c), with a similar loss of dopaminergic enervation in the striatum (Fig. 1b). Importantly, MPTP-injected RIP3ko mice were protected from dopaminergic neurodegeneration in the SN (Fig. 1a, c), which was accompanied by a tendency towards higher TH-positive staining in the striatum (Fig. 1b). These data suggest that RIP3 deletion attenuates neuronal death in the SN while only indirectly influencing loss of striatal dopaminergic enervation.

### RIP3ko leads to MPTP-driven exacerbation of striatal astrogliosis and GDNF replenishing

**Fig. 2:**
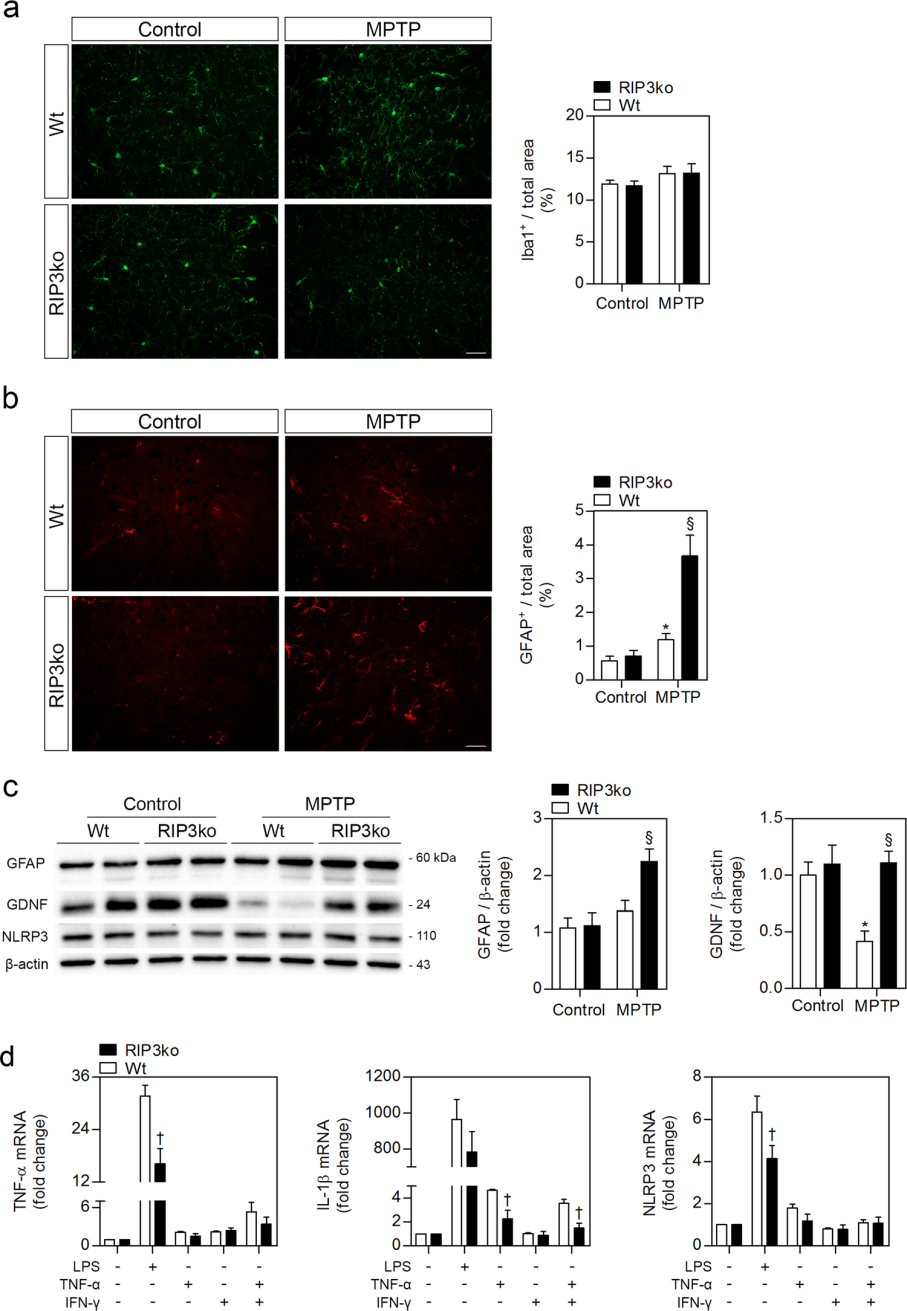
RIP3ko exacerbates striatal astrogliosis and replenishes GDNF protein levels in MPTP-injected RIP3ko mice when compared to Wt mice. **a** Representative images of Iba1-positive microglia in the striatum of control- and MPTP-injected mice from both genotypes and respective quantification. Scale bar, 100 μm. **b** Representative images of GFAP-positive astrocytes in the striatum of control- and MPTP-injected mice from both genotypes and respective quantification. Scale bar, 100 μm. **c** Representative WB of the SN of control– and MPTP-injected Wt and RIP3ko mice and respective densitometric analysis. Values represent mean ± SEM of 6-7 mice per group. *p < 0.05 from control Wt mice and ^§^*p* < 0.05 from MPTP-injected Wt mice. **d** Primary mixed glial cultures were exposed to 100 ng/ml LPS, 50 ng/ml TNF-α, 100 U/ml IFNγ or TNF-α + IFNγ for 24 h and pro-inflammatory gene expression was determined by qRT-PCR. Values represent mean ± SEM of three independent experiments. ^†^*p* < 0.05 from respective Wt group

MPTP mice models usually develop a fast inflammatory response that is started by microglia in the striatum, probably in response to MPP^+^-mediated damage to TH-positive fibers, followed by microgliosis in the SN^27^. Astrogliosis, on the other hand, develops later than microgliosis, and is sustained for longer periods of time, when microglia have already returned to a resting state^27–29^. Our results support this working hypothesis, since we could not detect any increase in Iba-1 immunostaining, a marker for microglia in the brain, in either striatum (Fig. 2a) or SN (Supplementary Fig. 1a), following MPTP intoxication for 30 days. Regarding astrogliosis, we also could not detect any alterations in GFAP immunostaining, a marker for astrocytes, in the SN (Supplementary Fig. 1b), suggesting total inflammation resolution. However, there was a significant increase in striatal GFAP-positive staining for MPTP-injected Wt animals, which was greatly exacerbated in MPTP-exposed RIP3ko mice (Fig. 2b). This was also confirmed by WB (Fig. 2c), thus suggesting a higher astrocytic response in MPTP-lesioned RIP3ko mice. Nevertheless, we could not detect mature TNF-α production in this brain region in any condition (data not shown), while the protein levels of NOD-, LRR- and pyrin domain-containing 3 (NLRP3), a cytosolic innate immune receptor involved in inflammasome activation^16^, also remained unaltered in all conditions (Fig. 2c). These data, along with lack of microglial activation, implicate other possible roles for elevated striatal astrogliosis besides inflammation. In fact, the protein levels of GDNF, a potent dopaminergic neurotrophic factor highly produced in the striatum and retrogradely transported by dopaminergic axons towards the cell bodies in the SN^30^, were greatly reduced in the striata of MPTP-exposed Wt mice, while being totally replenished in MPTP-exposed RIP3ko mice (Fig. 2c). In this regard, reactive astrocytes in dopamine-depleted striata following toxin-mediated lesions have been demonstrated to overexpress GDNF, which has subsequent neurotrophic properties in dopaminergic neurons^31–34^. Taken together, our observations support a possible neurotrophic role for the exacerbated astrogliosis seen in MPTP-injected RIP3ko mice.

To further compare the phenotypic effect of RIP3 deletion during inflammation, we exposed for 24 h primary cortical mixed glial cultures, composed mainly by astrocytes and ~10% microglia, to LPS, a strong pro-inflammatory stimulus, or TNF-α, IFN-γ, or a combination of the two, as a milder pro-inflammatory stimulus more related to PD^35,36^.

Regarding the expression of pro-inflammatory genes, RIP3ko cultures exposed to LPS expressed lower levels of TNF-α and NLRP3, along with a tendency towards lower IL-1β expression levels (Fig. 2d). Moreover, IL-1β mRNA levels were also significantly decreased following TNF-α or TNF-α + IFN-γ exposure (Fig. 2d). Overall, the expression of pro-inflammatory genes in RIP3ko cultures was reduced upon pro-inflammatory stimulation. However, despite a trend towards increased expression of GDNF following LPS and TNF-α + IFN-γ exposure, there was no differences between Wt and RIP3ko cultures, while protein levels of pro-GDNF remained similar in all conditions tested (Supplementary Fig. 1c), indicating that this protein is not significantly modulated. These analyses were performed 24 h post-exposure to pro-inflammatory stimuli to study the regulation of GDNF expression after the peak of the inflammatory response was overcome^37^. Our observations may be explained by the fact that GDNF expression is highly restricted to certain areas of the brain, suggesting that its regulation is probably dependent on the interaction between glial cells and specific neuronal types^30^.

### RIP3ko reduces markers of apoptosis in the SN

**Fig. 3:**
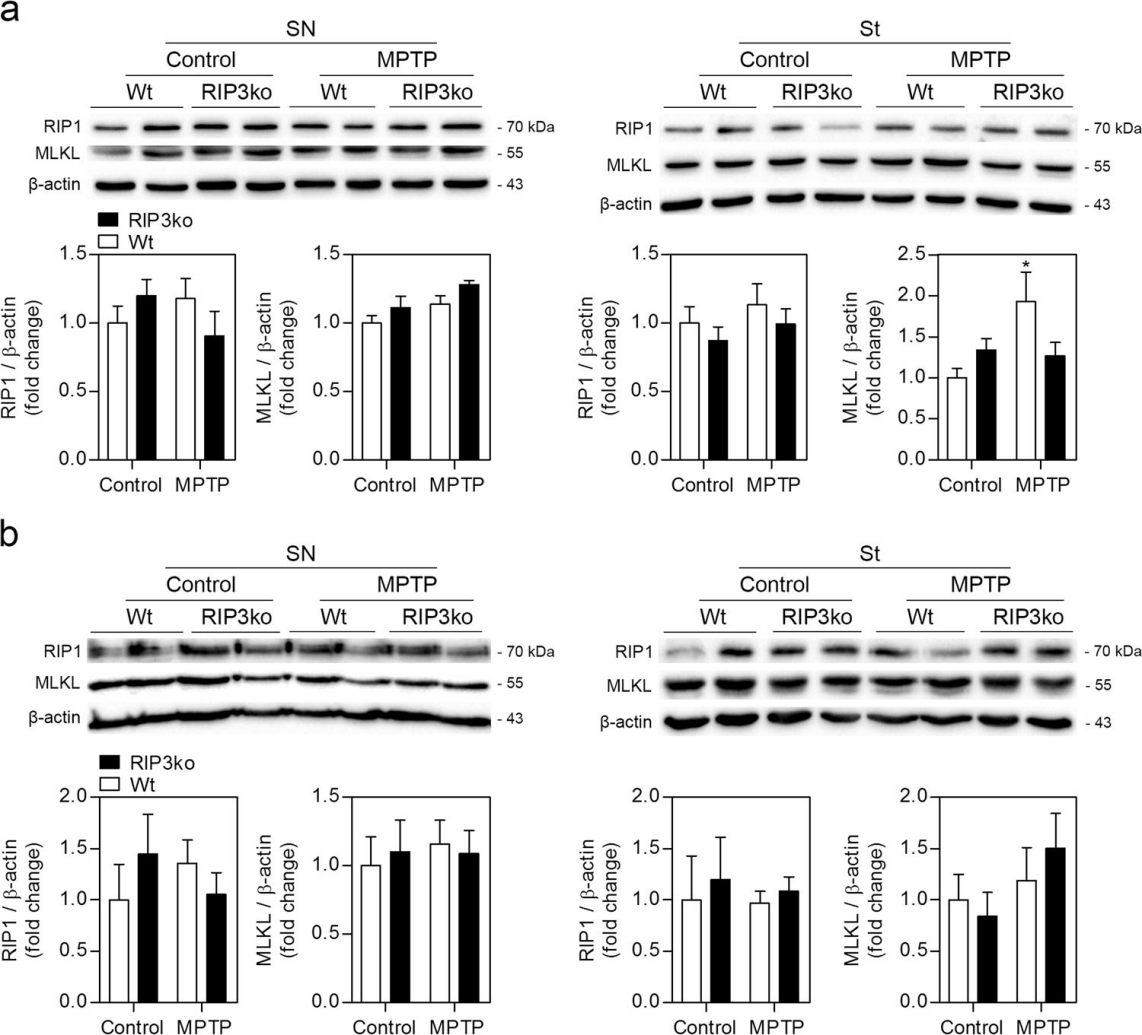
MPTP exposure does not increase RIP1 and MLKL recruitment into insoluble fractions. Representative WB of soluble (**a**) and insoluble (**b**) protein fractions from SN and striatum of control– and MPTP-injected Wt and RIP3ko mice and respective densitometric analysis. Values represent mean ± SEM of 6–7 mice per group. *p < 0.05 from control Wt mice

**Fig. 4:**
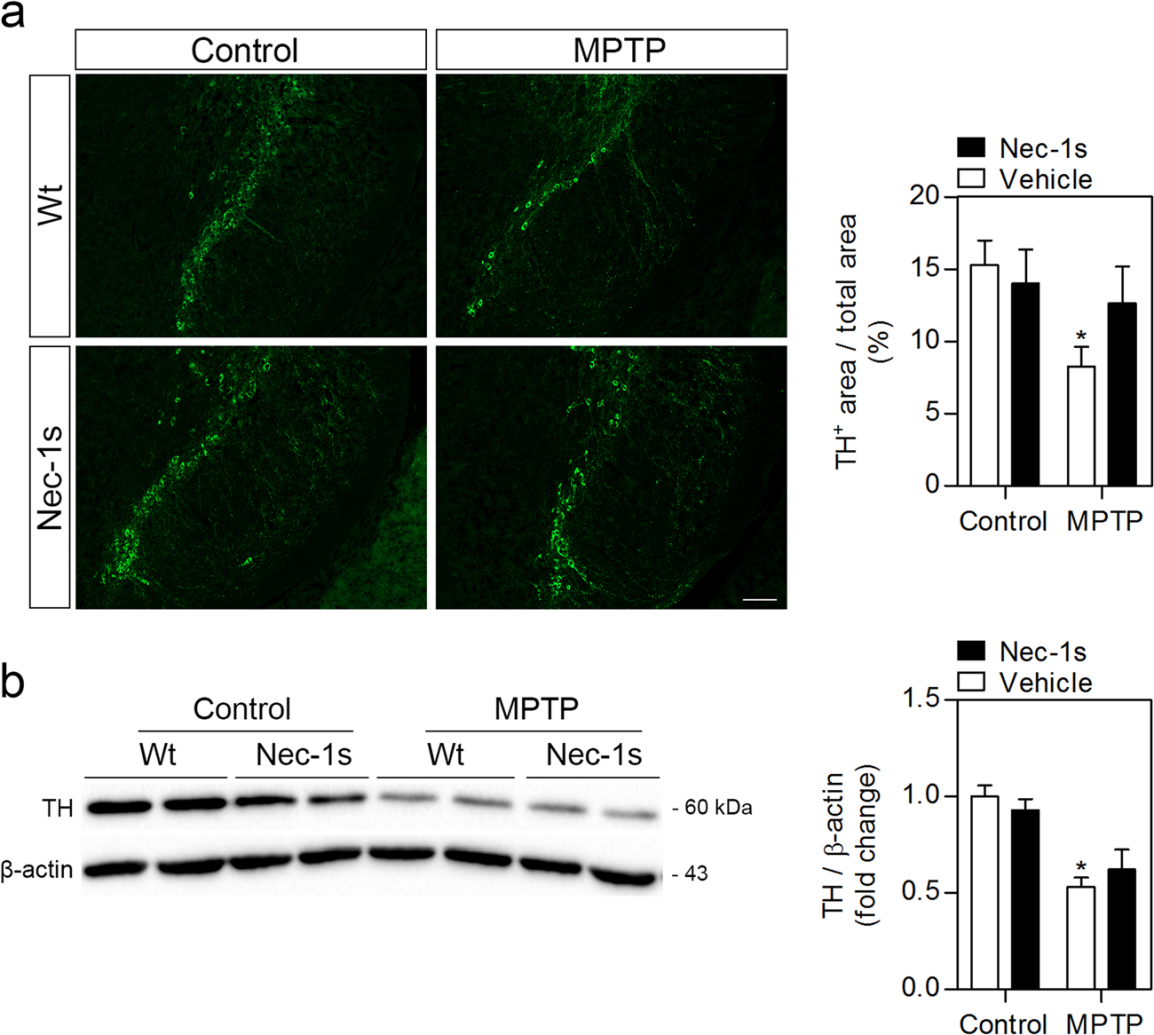
Nec-1s does not significantly protect from MPTP-driven dopaminergic neuronal loss. **a** Representative images of TH-positive immunostaining in the SN from control- and MPTP-injected mice treated with either vehicle or Nec-1s and respective quantification. Scale bar, 100 μm. **b** Representative WB of TH protein levels in the SN of control– and MPTP-injected mice treated with either vehicle or Nec-1s and respective densitometric analysis. Values represent mean ± SEM of 6–7 mice per group. *p < 0.05 from control Wt mice

Due to the role of RIP3 in necroptosis, we hypothesized that protection from MPTP-mediated dopaminergic loss in RIP3ko animals was due to inhibition of necroptosis. Therefore, several markers of necroptotic commitment in the SN and striatum of Wt mice were measured at 4, 6 and 30 days after MPTP exposure, namely RIP3-dependent MLKL phosphorylation and RIP1/MLKL sequestration in insoluble fractions, an indication of necrosome assembly^8,13,14^. However, we could not detect any MLKL phosphorylation by WB in either soluble or insoluble fractions, or by immunohistochemistry in the SN. Moreover, soluble RIP1 and MLKL protein levels also remained stable at 4 and 6 days post-injection (Supplementary Fig. 2a), and at 30 days (Fig. 3a), with the exception of MLKL in the striatum, which was significantly increased in MPTP-treated Wt mice. RIP1 and MLKL were not enriched in the insoluble fractions obtained from the striata and midbrains from these animals at either 4 and 6 days post-injection (Supplementary Fig. 2b), or at 30 days (Fig. 3b). These data suggest that necroptosis may not play a significant role in dopaminergic neurodegeneration at the time-points observed, although we cannot exclude a role for this form of cell death between 6 and 30 days. However, the peak of neurodegeneration in our MPTP model may occur around 7 days after MPTP injection, thus arguing against this possibility^19^. Finally, Nec-1s administration did not offer full protection from dopaminergic neurodegeneration in the SN (Fig. 4a, b). Of note, a similar regimen of Nec-1s administration has been previously reported to protect from neurodegeneration in a sub-chronic MPTP mouse model^7^, further supporting the hypothesis that necroptosis may not be involved in cell death in the MPTP model used in this study. Nevertheless, Nec-1s presents a very short t_1/2_ in vivo, indicating that the administration regimen used may not be sufficient to completely block RIP1-dependent necroptosis in all experimental settings^8^.

**Fig. 5:**
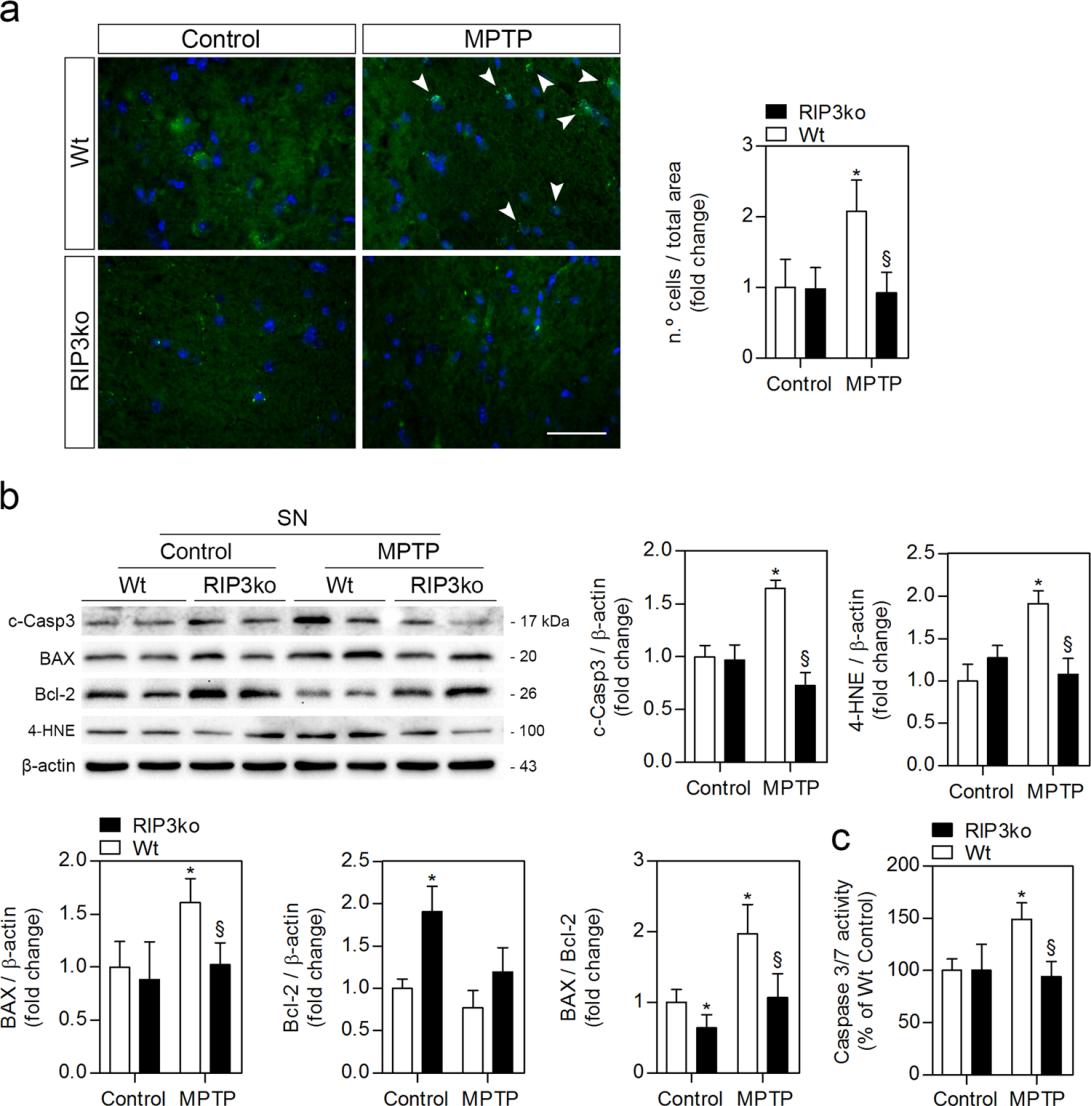
RIP3ko protects from MPTP-driven DNA fragmentation, 4-HNE accumulation, caspase-3 activation and increased BAX expression detected in Wt mice. **a** Representative images of TUNEL-positive apoptotic nuclei in the SN of control- and MPTP-injected mice from both genotypes and respective quantification. Scale bar, 40 μm. **b** Representative WB of soluble proteins from SN of controls and MPTP-injected mice from both genotypes and respective densitometric analysis. **c** BAX/Bcl-2 ratio and caspase-3/7 activity were determined in protein samples from SN of control– and MPTP-injected mice from both genotypes. Values represent mean ± SEM of 6–7 mice per group. *p < 0.05 from control Wt mice and ^§^*p* < 0.05 from MPTP-injected Wt mice. c-Casp3: cleaved caspase-3

Interestingly, MPTP-injected Wt mice presented higher number of TUNEL-positive nuclei in the SN, a possible indication of apoptotic-dependent DNA fragmentation (Fig. 5a). Moreover, we could still detect increased cleaved caspase-3 and caspase-3/7 activities in the midbrains of MPTP-injected Wt mice (Fig. 5b, c). BAX protein levels were also elevated in these animals, thus leading to a higher BAX/Bcl-2 ratio (Fig. 5c), which may be indicative that mitochondrial-dependent apoptosis does still play a role in cell loss in the midbrain at this time-point^3^. Of note, MPTP-injected RIP3ko mice were significantly protected against DNA fragmentation, caspase-3 activation and BAX overexpression, suggesting that deletion of RIP3 may influence cell death in a non-necroptotic manner. Intriguingly, RIP3ko mice appear to have increased Bcl-2 levels in the midbrain at basal levels, which further supports our results (Fig. 5b). This phenotype was only specific to the midbrain, since Bcl-2 levels remained unaltered in the striatum, cortex, cerebellum, spleen and liver of RIP3ko mice, an indication that this phenotype depends on cell type/location (Supplementary Fig. 3).

Since MPTP-mediated toxicity is dependent on increased reactive oxygen species (ROS) production, we evaluated the production of 4-HNE, a toxic product of ROS-dependent peroxidation of arachidonic acid in cell membranes that can interact with proteins forming adducts^38,39^. Importantly, 4-HNE levels are increased in the SN and cerebrospinal fluid of PD patients, as well as in the brainstem of an MPTP mouse model similar to the one used here^38,39^. As expected, 4-HNE levels doubled in the SN of MPTP-injected Wt mice but were reverted to control levels in MPTP-exposed RIP3ko mice (Fig. 5b). These results suggest that RIP3 deletion may reduce MPTP-driven oxidative damage, which may be either upstream or downstream of the induction of intrinsic apoptosis.

### RIP3ko primary cortical neurons are protected from MPP^+^-driven cell death

**Fig. 6:**
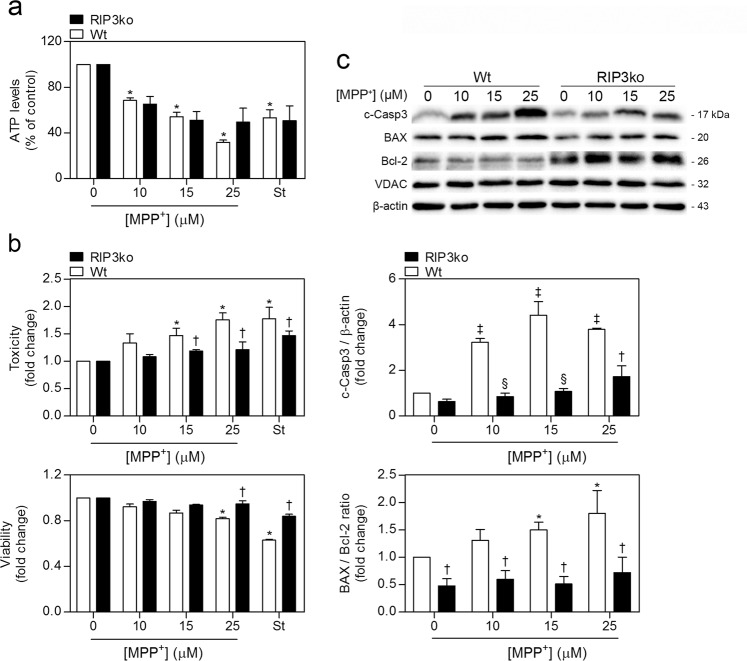
RIP3ko attenuates MPP^+^-mediated cell death, caspase-3 activation and elevation of the BAX/Bcl-2 ratio in primary cortical neurons. Primary cortical neurons were exposed to 0, 10, 15, and 25 μM MPP^+^ or 100 nM staurosporine and total ATP levels (**a**), toxicity/viability (**b**) and total protein levels (**c**) were determined after 24 h. Values represent mean ± SEM of four independent experiments. *p < 0.05 and ^‡^*p* < 0.01 from control Wt group; ^†^*p* < 0.05 and ^§^*p* < 0.01 from respective Wt group. c-Casp3: cleaved caspase-3

To further dissect the role of RIP3 ablation in neuronal death, we exposed primary cortical neurons at 7DIV from Wt and RIP3ko mice to increasing concentrations of MPP^+^, the toxic metabolite of MPTP, and staurosporine. MPP^+^ is a mitochondrial complex-I inhibitor, which leads to a sharp decline in ATP production and ROS accumulation^40^. As expected, ATP levels decreased in an MPP^+^-dose dependent fashion, which was independent on the genotype of the primary cultures (Fig. 6a). Staurosporine, a known inducer of ROS-dependent apoptosis in cultured neurons^41^, led to a reduction of ~50% in total ATP levels, similar to the results obtained for 15 μM MPP^+^ (Fig. 6a).

However, although the decline in ATP levels remained unaltered in RIP3ko cultures following MPP^+^ exposure, deletion of RIP3 significantly reduced toxicity and increased viability of primary neurons after exposure to 15 and 25 μM MPP^+^, as well as after exposure to 100 nM staurosporine (Fig. 6b).

Once again, no MLKL phosphorylation was detected in MPP^+^-exposed Wt neurons, along with no significant alterations in MLKL or RIP1 total protein levels (Supplementary Fig. 4). These results support those observed in vivo, further lessening the role of necroptosis in MPP^+^-dependent cell death in our rodent models. Moreover, a strong elevation up to 4-fold in cleaved caspase-3 was evident in MPP^+^-treated Wt neurons, which was mostly abrogated in RIP3ko neurons (Fig. 6c). We could not detect any increase in cleaved, and therefore active, caspase-8, further supporting a role for intrinsic, and thus mitochondrial-dependent apoptosis, rather than extrinsic apoptosis, which typically depends on caspase-8 activation^3^. Interestingly, the BAX/Bcl-2 ratio is also consistently higher in Wt neurons than in RIP3ko, further implying mitochondrial dysfunction in MPP^+^-driven cell death (Fig. 6c).

## Discussion

Initially, we had hypothesized that RIP3 deletion could attenuate MPTP-driven dopaminergic neurodegeneration by preventing damaged neurons in the SN from undergoing necroptosis. Unexpectedly, although MPTP-injected RIP3ko mice were significantly protected from neuronal loss in the SN, no markers of necroptosis were detected at either 4, 6 or 30 days. Moreover, Nec-1s administration did not fully protect from dopaminergic neurodegeneration in the SN, further indicating that necroptosis is possibly not crucially involved in RIP3ko-dependent mitigation of MPTP pathology in our in vivo model. This same regimen of Nec-1s administration was previously described as protective against neurodegeneration in a sub-chronic MPTP mouse model (20 mg/kg MPTP (i.p.) injected daily for 5 days)^7^. Regarding these discrepant results, it is possible that a more prolonged exposure to low MPTP doses may alter cell death pathways^40,42^. However, it has been demonstrated that apoptosis contributes to dopaminergic neuronal loss in several MPTP mouse models, including the sub-chronic model and, indirectly via JNK activation, the sub-acute model used here^19,42–44^. Apoptosis in these models is dependent on mitochondrial oxidative stress and BAX, a proapoptotic member of the Bcl-2 family, which is upregulated in SN dopaminergic neurons upon MPTP challenge, along with decreased protein levels of the anti-apoptotic Bcl-2, an antagonist of BAX function^42,45,46^. Moreover, BAX ablation in mice strongly protects against MPTP-mediated neurodegeneration^45^. In turn, this increase in BAX/Bcl-2 ratio facilitates cytochrome *c* release, which then leads to activation of caspase-9 and its downstream target caspase-3^47,48^. Of note, higher BAX levels and increased caspase-3 and -9 activities have also been reported in the SN of PD patients, along with markers of oxidative damage such as 4-HNE^39,49,50^. Overall, these results fully support a role for apoptosis in MPTP-driven cell death and PD. Here, we detected elevated TUNEL-positive nuclei, BAX protein levels and caspase-3 activation, along with higher 4-HNE-protein adducts in the SN of MPTP-injected Wt mice, with no caspase-8 cleavage. Moreover, all these MPTP-dependent alterations were abrogated in MPTP-lesioned RIP3ko mice, which also presented higher Bcl-2 protein levels in control conditions in the SN, thus further contributing to a lower BAX/Bcl-2 ratio. Interestingly, Bcl-2 is not only an integral mitochondrial protein that limits cytochrome *c* release and caspase activation, but also directly contributes to limit oxidative damage^41^. In accordance, Bcl-2 overexpression in mice attenuates MPTP-mediated neurotoxicity in vivo and in vitro^51–53^. Interestingly, increased Bcl-2 levels were detected specifically in the midbrains of RIP3ko mice, and not in other brain regions and peripheric organs. Moreover, necroptosis in multiple settings may lead to cell death by recruiting the oligomerization of BAX/ Bcl-2-antagonist/killer 1 (BAK1), a process typically associated with apoptosis that leads to mitochondrial outer membrane permeabilization^54–57^. These data hint at the possibility that there may be still undescribed links between the necroptotic machinery and proteins from the Bcl-2 family.

Importantly, our in vitro results closely mimicked the results observed in vivo, since RIP3ko primary neurons were significantly protected from MPP^+^-driven toxicity, probably due to decreased caspase-3 activation and a lower BAX/Bcl-2 ratio^6,54^. RIP3ko-mediated protection was independent of the reposition of decreasing ATP levels following MPP^+^ exposure, which remained similar between Wt and RIP3ko cultures. Interestingly, RIP3ko neurons were significantly protected against staurosporine-induced apoptosis, which is also driven by mitochondrial ROS production and subsequent dysfunction, further supporting an effect for RIP3 deletion in mitochondrial protection^41^.

The mechanisms of MPP^+^-driven cell death in vitro are highly controversial. Multiple papers report detection of apoptotic features along with significant protection from MPP^+^ toxicity in primary neuronal cultures following caspase inhibition^52,58,59^, while others have reported absence of such markers or protection upon caspase inhibition^60–62^. These differences probably arise from different MPP^+^ concentrations and culture conditions. The extent of MPP^+^-driven ATP depletion is probably the most important factor for commitment to a specific RCD, since apoptosis is ATP-dependent. In this regard, absence of apoptotic features during MPP^+^-induced cell death has been reported despite cytochrome *c* release from mitochondria, but this form of cell death shifted to typical apoptosis following ATP supplementation^62^. In our experimental design, exposure of primary cortical neurons up to 15 μM MPP^+^ led to a reduction of ~50% in ATP levels, similar to the reduction induced by staurosporine, a known apoptotic stimulus, which suggests that these are apoptotic permissive conditions.

Surprisingly, caspase-8 activation has been detected in MPP^+^-exposed primary neuronal cultures, but caspase inhibition shifted cell death from apoptosis to necrosis^61^. Nevertheless, caspase-8 may be activated as an amplification step of the caspase cascade downstream of caspase-9, following MPP^+^-driven cytochrome *c* release^48^. We could not detect cleaved, and therefore active, caspase-8 in our in vitro experiments, further supporting a primary role for mitochondrial dysfunction in MPP^+^-dependent cell death. In turn, lack of caspase-8 involvement argues against a possible reduction in Ripoptosome formation, and therefore caspase-8-dependent apoptosis, when RIP3 is deleted in our experimental settings^15^. Overall, our results hint at still undescribed roles for RIP3 in mitochondrial dysfunction and cell death.

RIP3 deletion affords more protection than MLKL deficiency in mouse models of renal injury, metastatic tumor, TNF-α challenge, arthritis and infection, supporting other non-necroptotic roles for RIP3^16,63–65^. Interestingly, several of these functions may be linked to inflammation. In fact, dendritic cells from RIP3ko mice develop a dampened inflammatory response following LPS exposure^17^. RIP3 can also mediate inflammasome activation and subsequent IL-1β maturation in a MLKL-independent fashion under certain conditions^16^. Our results also point towards an inflammatory role for RIP3 independent of necroptosis, since RIP3ko mixed glial cultures displayed lower mRNA levels of TNF-α, NLRP3 and, IL-1β following pro-inflammatory stimulation. Moreover, MPTP-injected RIP3ko mice presented higher striatal GFAP immunostaining than MPTP-injected Wt mice, suggesting prolonged astrogliosis in this brain region. However, microgliosis was not detected in these MPTP-exposed animals, suggesting a non-inflammatory phenotype for these astrocytes. GDNF protein levels, which were massively reduced in MPTP-injected Wt mice, were replenished in the striata of MPTP-injected RIP3ko mice. Of note, GDNF reposition may be mediated by the reactive astrocytes detected, thus supporting dopaminergic viability^31–33^. Moreover, similarly to our results, RIP3 ablation was shown not only to protect reactive astrocytes from undergoing necroptosis following spinal cord injury, but also rescued the neurotrophic functions of these astrocytes as detected by increased GDNF expression, further supporting a role for RIP3 in astrocytic function^66^. Overall, our results point towards a more neurotrophic role for the reactive astrocytes in the striatum of MPTP-injected RIP3ko mice, suggesting that RIP3 may play a role during glial activation. It is also possible that the higher dopaminergic viability in RIP3ko mice may contribute to shift astrocytic phenotypes towards more neurotrophic roles, although only a tendency towards higher striatal TH-positive fiber content were detected in MPTP-injected RIP3ko mice.

Overall, our results hint at possible non-necroptotic roles for RIP3 in inflammation and MPP^+^-driven apoptosis. Here, RIP3 ablation dampened the expression of pro-inflammatory mediators in primary mixed glial cultures and rescued GDNF protein levels in the striatum of MPTP-injected mice, while protecting from MPTP/MPP^+^-driven dopaminergic degeneration along with caspase-3 activation and lower BAX/Bcl-2 ratio in vivo and in vitro. In this regard, further work is needed to understand the kinase-dependent vs. scaffolding functions of RIP3 in PD-driven degeneration, which will impact on the therapeutic targeting of RIP3. However, it is probable that necroptosis is involved in other paradigms of MPTP-driven neurotoxicity and PD progression. In this regard, a full characterization of neurodegeneration forms in cohorts of etiologically diverse PD patients would be instrumental.

## Supplementary information

Supplemental material

Supplementary table
