# Supplementary material for: Ablation of RIP3 protects from dopaminergic neurodegeneration in experimental Parkinson’s disease
Source: Cell Death Dis. 2019 Nov 5;10(11):840. doi: 10.1038/s41419-019-2078-z (PMC6831575; doi:10.1038/s41419-019-2078-z)

**Supplementary Figure Legends**

**Supp. Fig. 1** MPTP-injected mice do not develop sustained microgliosis and astrogliosis in the SN. (**a**) Representative images of Iba1-positive microglia in the SN of control- and MPTP-injected mice from both genotypes and respective quantification. *Scale bar*, 100 μm. (**b**) Representative images of GFAP-positive astrocytes in the SN of control- and MPTP-injected mice from both genotypes and respective quantification. *Scale bar*, 100 μm. (**c**) Primary mixed glial cultures were exposed to 100 ng/ml LPS, 50 ng/ml TNF-α, 100 U/ml IFNγ or TNF-α+IFNγ for 24 h and GDNF mRNA levels and pro-GDNF protein levels were determined by qRT-PCR and WB respectively. Values represent mean ± SEM of 3 independent experiments. R3ko: RIP3ko

**Supp. Fig.** **2** MPTP exposure for 4 and 6 days does not increase RIP1 and MLKL recruitment into insoluble fractions. Representative WB of soluble **(a)** and insoluble **(b)** protein fractions from SN and striatum of control– and MPTP-injected Wt and RIP3ko mice.

**Supp. Fig. 3** Bcl-2 protein levels are similar between Wt and RIP3ko mice in the striatum, cortex, cerebellum, liver and spleen. Representative WB of total protein samples from striatum, cortex, cerebellum, liver and spleen of Wt and RIP3ko mice. Values represent mean ± SEM of 3 mice per group.

**Supp. Fig. 4** RIP1 and MLKL protein levels remain constant following MPP^+^ exposure in both Wt and RIP3ko neurons. Representative WB of total protein samples from Wt and RIP3ko primary cortical neurons exposed to 0, 10, 15 and 25 μM MPP^+^ for 24 h. Values represent mean ± SEM of 4 independent experiments.

**
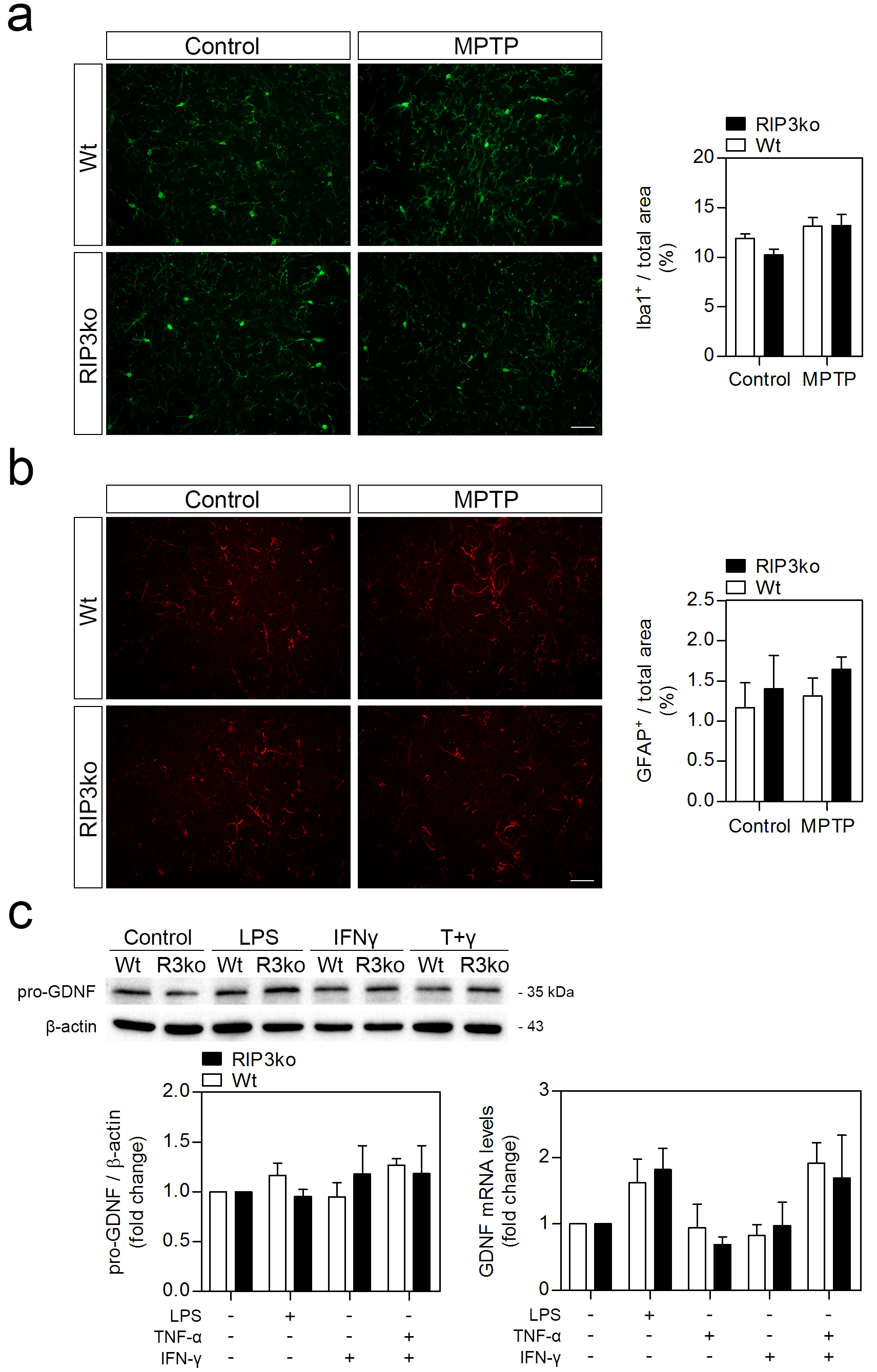
SUPPL. FIGURE 1**

**SUPPL. FIGURE 2**


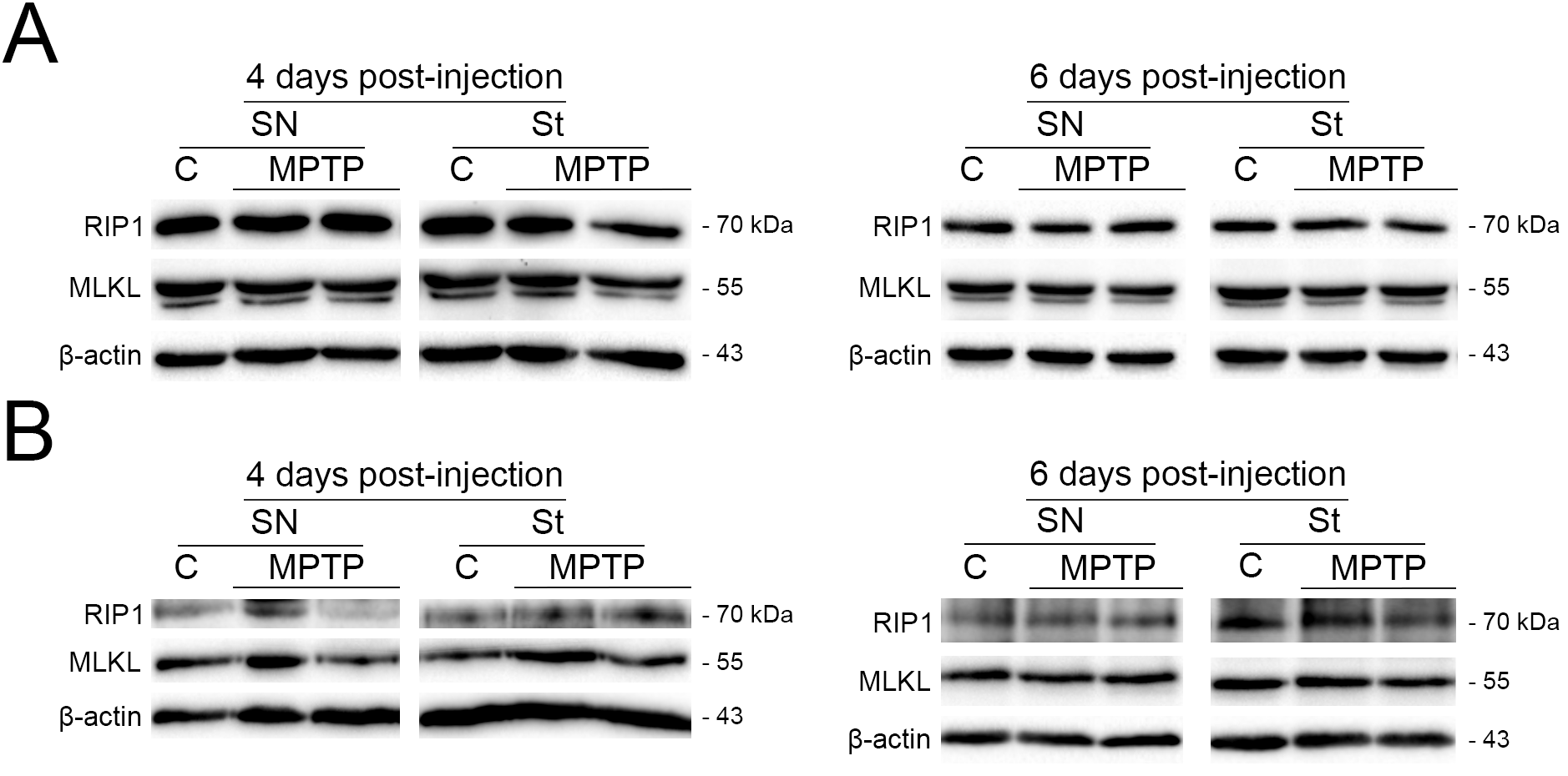


**SUPPL. FIGURE 3**


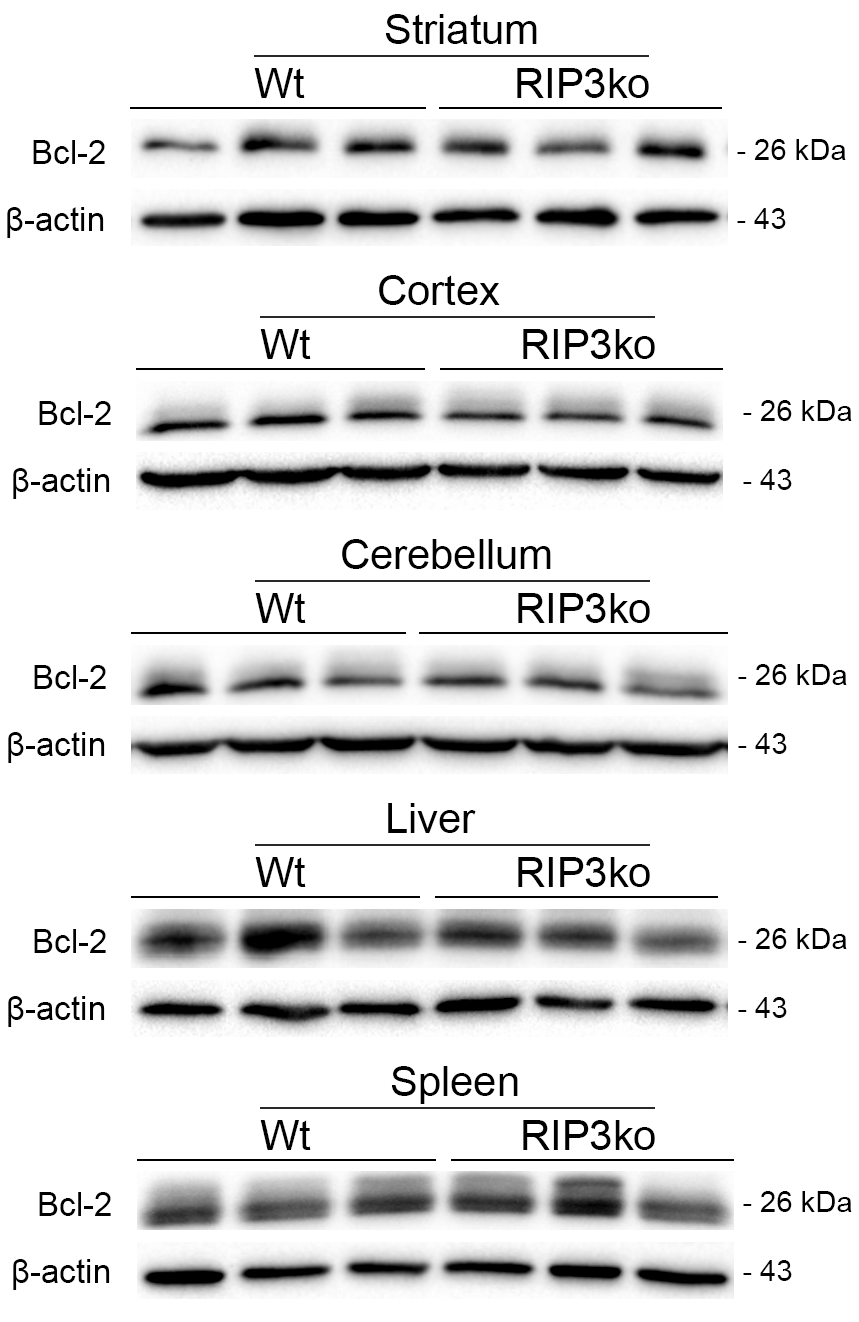


**SUPPL. FIGURE 4**


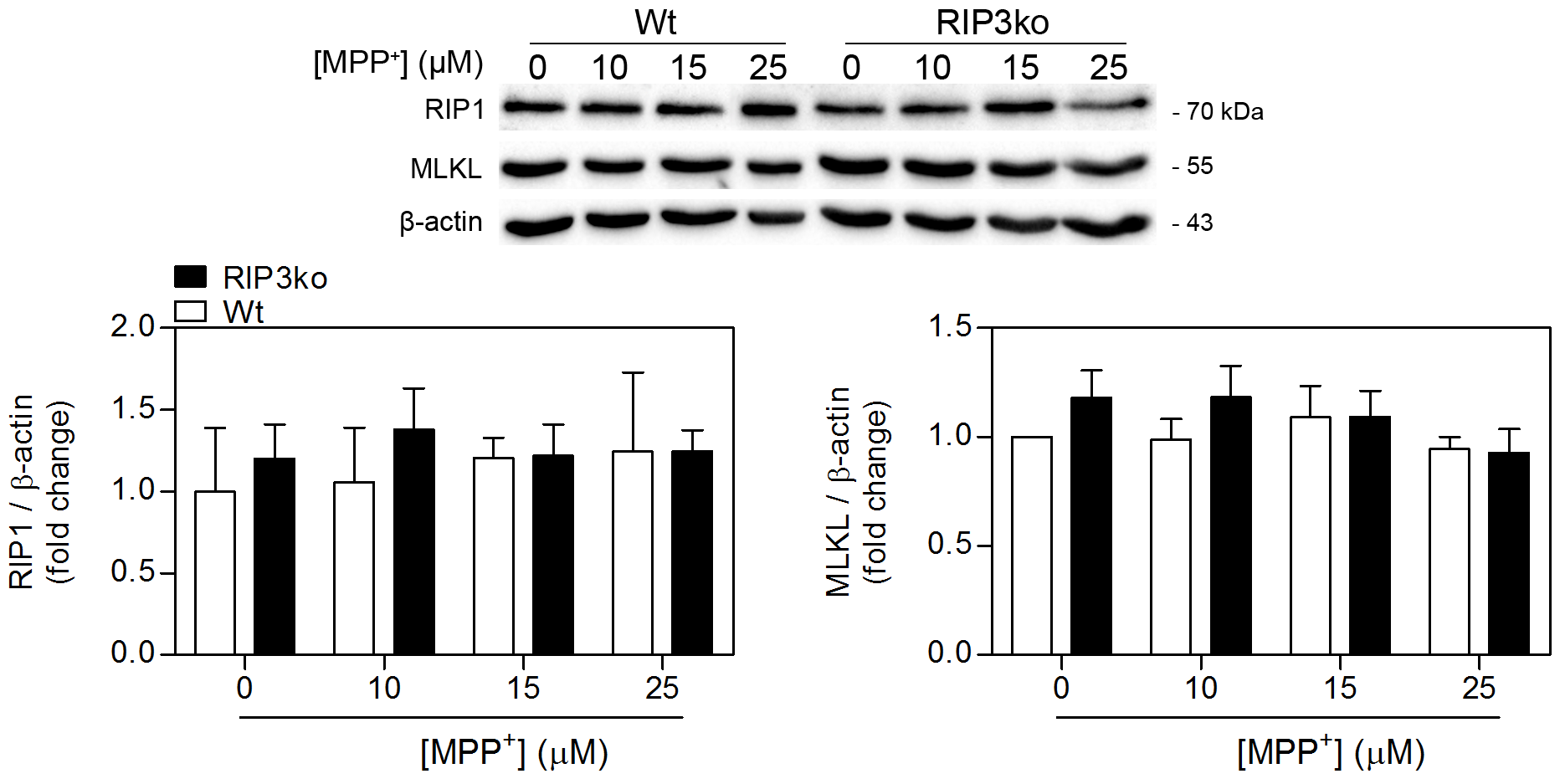

Supplement: Supplementary file 1 — Supplemental material [file 41419_2019_2078_MOESM1_ESM.docx]
