# Supplementary material for: Ablation of RIP3 protects from dopaminergic neurodegeneration in experimental Parkinson’s disease
Source: Cell Death Dis. 2019 Nov 5;10(11):840. doi: 10.1038/s41419-019-2078-z (PMC6831575; doi:10.1038/s41419-019-2078-z)
Supplement: Supplementary file 2 — Supplementary table [file 41419_2019_2078_MOESM2_ESM.docx]

| **Supplementary Table 1** Primer sequences used to amplify indicated mouse cDNAs | | |
| --- | --- | --- |
|  | **Sense primer (5’-3’)** | **Antisense primer (5’-3’)** |
| **HPRT** | GGTGAAAAGGACCTCTCGAAGTG | ATAGTCAAGGGCATATCCAACAACA |
| **TNFα** | AGGCACTCCCCCAAAAGATG | TGAGGGTCTGGGCCATAGAA |
| **IL-1β** | TGCCACCTTTTGACAGTGATG | TGATGTGCTGCTGCGAGATT |
| **NLRP3** | AGAGCCTACAGTTGGGTGAAATG | CCACGCCTACCAGGAAATCTC |
